# Neurofilament Light in CSF and Plasma Is a Marker of Neuronal Damage in HTLV-1–Associated Myelopathy and Correlates With Neuroinflammation

**DOI:** 10.1212/NXI.0000000000001090

**Published:** 2021-10-05

**Authors:** Carolina Rosadas, Henrik Zetterberg, Amanda Heslegrave, Jana Haddow, Mina Borisova, Graham P. Taylor

**Affiliations:** From the Section of Virology (C.R., G.P.T.), Department of Infectious Disease, Imperial College London; UK Dementia Research Institute at UCL (H.Z., A.H., M.B.); Department of Neurodegenerative Disease (H.Z., A.H., M.B.) at UCL Institute of Neurology, London, UK; Clinical Neurochemistry Laboratory (H.Z.), Sahlgrenska University Hospital; Department of Psychiatry and Neurochemistry (H.Z.), Institute of Neuroscience and Physiology, the Sahlgrenska Academy at the University of Gothenburg, Mölndal, Sweden; and National Centre for Human Retrovirology (J.H., G.P.T.), St. Mary's Hospital, Imperial College Healthcare NHS Trust, London, UK.

## Abstract

**Background and Objectives:**

To evaluate the usefulness of CSF and plasma neurofilament light (Nf-L) as a biomarker for human T-cell lymphotropic virus type 1 (HTLV-1)-associated myelopathy (HAM).

**Methods:**

Nf-L, CXCL10, and neopterin were measured by ELISA in 83 CSF samples obtained from 49 individuals living with HTLV-1/2. Plasma Nf-L was also measured by single molecule array. Results were correlated with duration of disease, age, mobility, CSF cell counts, CSF protein, and HTLV-1 proviral load.

**Results:**

Nf-L was detected in all CSF samples (median [range] = 575 [791.8–2,349] pg/mL) and positively correlated with markers of inflammation (CXCL10 (r = 0.733), neopterin (r = 0.499), cell count (r = 0.403), and protein levels (r = 0.693) in CSF; *p* < 0.0015). There was an inverse correlation between Nf-L and duration of disease (r = −0.584, *p* < 0.0001). Wheelchair-dependent patients had high concentrations of markers of inflammation and neuronal damage. Concentrations of CXCL10, neopterin, and Nf-L remained elevated in follow-up samples (mean follow-up 5.2 years). Nf-L in plasma correlated with concentration of Nf-L, neopterin, CXCL10, and protein in CSF.

**Conclusions:**

Nf-L in plasma and CSF has potential to be used as a biomarker of disease activity in HAM. Neuronal damage seems to be more intense early in disease but persists long term. Wheelchair-dependent patients have ongoing neuroinflammation.

Human T-cell lymphotropic virus type 1 (HTLV-1) infects for life at least 5–10 million individuals.^1^ In approximately 3% of infected individuals, HTLV-1 causes spinal cord inflammation, resulting in HTLV-1–associated myelopathy (HAM). HAM pathogenesis is usually characterized by an initial inflammatory phase, followed by atrophy and neurodegeneration.^2^ The clinical course, although diverse, from mildly impaired mobility after decades to wheelchair dependency in weeks, greatly affects the patient's autonomy and quality of life.^3^ Some asymptomatic individuals also have discreet neurologic signs suggesting limited CNS damage.^4^ Cognitive dysfunction and encephalitis have also been reported with HTLV-1, indicating that the damage is not restricted to the spinal cord.^5^ Recently, a classification of disease severity was proposed based on neopterin and CXCL10 concentrations in CSF which correlated with very slow, slow, or rapid progression.^6^ CXCL10 has been associated with HAM pathogenesis via a positive feedback loop involving interferon-induced astrocyte activation, upregulation of CXCL10, and recruitment of inflammatory cells to the spinal cord. However, markers of neuronal damage have not been comprehensively studied in HAM.^2^ The identification of markers of minor or ongoing neurologic damage is important to identify incipient lesions to guide early therapeutic interventions and to better stratify patients in future therapeutic trials. Neurofilaments are structural proteins that are only expressed in neurons. Neurofilament light (Nf-L) is being increasingly used as a marker of neuronal damage.^7^ In this study, markers of inflammation and neuronal damage were measured in CSF and plasma to verify the potential of Nf-L as a biomarker for HAM.

## Methods

Stored paired ethylenediamine tetraacetic acid plasma and CSF (n = 83) obtained from 49 patients attending the National Centre for Human Retrovirology, St. Mary's Hospital, London, were included in the study: 41 patients with HAM; 3 HTLV-1 asymptomatic carriers; 1 patient with small fiber neuropathy; 3 coinfected with HIV-1, of which 2 had HAM; and 1 HTLV-2 asymptomatic carrier. Nineteen patients with HAM had samples at 2–6 different time points, mean follow-up 5.2 years (range 0.5–11 years). Clinical data consisting of age, sex, duration of symptoms, and disease severity, as assessed by walking aid usage, were also analyzed.

HTLV-1 proviral load was determined by real-time PCR.^8^ Routine CSF analysis included total protein and cell counts. All CSF markers were measured by ELISA, according to manufacturers' instructions. CSF samples were assayed undiluted for neopterin (IBL International, Germany), whereas 1:2 and 1:10 dilutions were used for Nf-L and CXCL10, respectively (Abcam and Uman Diagnostics). Nf-L was quantified in undiluted plasma by ELISA (n = 45) and by the highly sensitive, single molecule array (SIMOA, Quanterix, Billerica, MA) assay (n = 23). All samples were assayed in duplicate, and the mean value was reported.

### Standard Protocol Approvals, Registrations, and Patient Consents

The samples had been donated by patients following written informed consent to the Communicable Diseases Research Tissue Bank National Research Ethics Service reference 15/SC/0089 or were residual samples after routine clinical investigations were complete with linked-anonymized clinical data provided by the clinical service.

### Data Availability

Deidentified data will be shared on request from a qualified investigator.

## Results

Demographics, clinical characteristics, and routine laboratory measures are summarized in the supplementary eTable, links.lww.com/NXI/A605. Neopterin (median 21.9 nmol/L, range 1.1–111 nmol/L) was detected in every CSF sample. CXCL10 (median 1,296 pg/mL, range 0–12,429 pg/mL) was not detected in 2 asymptomatic carriers and in 1 patient with HAM, who was still walking unaided 3 years after disease onset with no deterioration in 10-m time walk.

Nf-L was detected in all CSF samples (n = 73) (median 575 pg/mL, range 21–26,059 pg/mL). According to the manufacturer's reference range and accounting for patient's age, all 4 (100%) asymptomatic carriers had normal Nf-L concentrations, whereas 37% of CSF samples from patients with HAM were high. Median plasma Nf-L was 10.5 ranging from 4.3 to 154.5 pg/mL.

**Figure 1:**
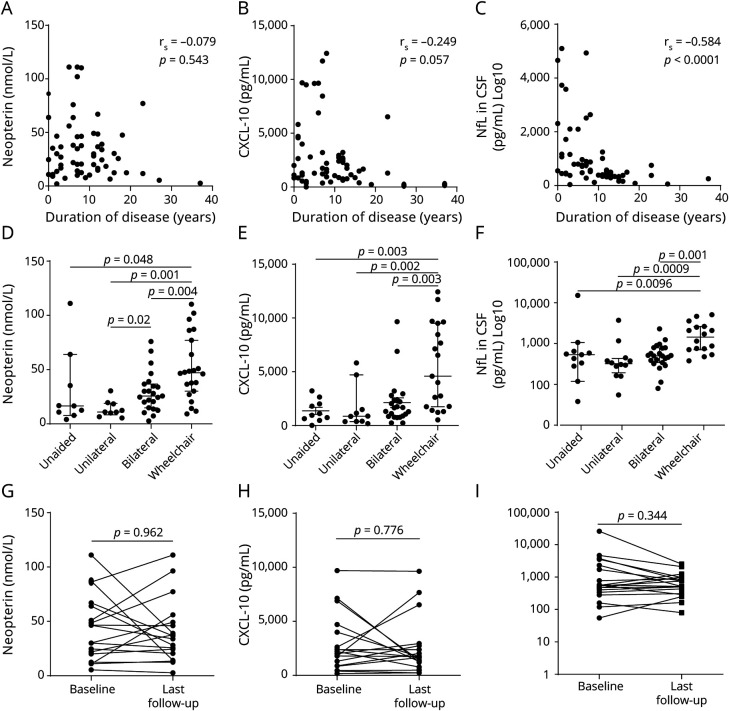
Markers of Inflammation and Neuronal Damage in Patients With HAM Correlation between duration of disease (years) and neopterin concentration (nmol/L) (A), CXCL10 (pg/mL) (B), and Nf-L (pg/mL) (C) in CSF measured by ELISA. Correlation coefficient calculated using the Spearman test and *p* values are shown. Each dot represents 1 sample; levels of neopterin (nmol/L) (D), CXCL10 (pg/mL) (E), and Nf-L (F) in CSF measured by ELISA according to the disease severity, evaluated by walking aid need (unaided, unilateral, bilateral, and wheelchair); Variation of CSF biomarkers over time in patients with HAM (G-I). The Mann-Whitney test was used to compare groups, and *p* values are shown when statistically significant (*p* > 0.05). HAM = human T-cell lymphotropic virus type 1–associated myelopathy; Nf-L = neurofilament light.

**Figure 2:**
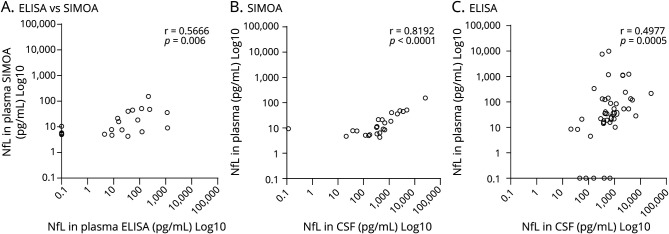
Correlation Between Neurofilament Light (pg/mL) in Plasma Measured by SIMOA and ELISA (A) and Between Plasma and CSF Samples According to the Different Techniques: SIMOA (B) and ELISA (C) Each dot represents 1 sample. Correlation coefficient (rs) was calculated using the Spearman test, and *p* values are shown. SIMOA = single molecule array.

There were significant positive correlations between the markers of inflammation (CSF protein, CSF cell count, neopterin, and CXCL10), with the exception of CSF cell count with CXCL10 (Table 1). Nf-L in CSF correlated positively with all markers of inflammation and negatively with duration of disease (Figure 1, A–C). There was no correlation between Nf-L and age. Nf-L in plasma had a strong correlation with Nf-L in CSF and with markers of inflammation (Table 1). Twenty-two plasma samples were assayed for Nf-L by ELISA and by SIMOA. The overall correlation was moderate (r = 0.566, *p* = 0.006), although discrepant results (higher concentrations of Nf-L in plasma when measured by ELISA than in CSF) were observed in 8.8% of patients (Figure 2).

**Table 1:**
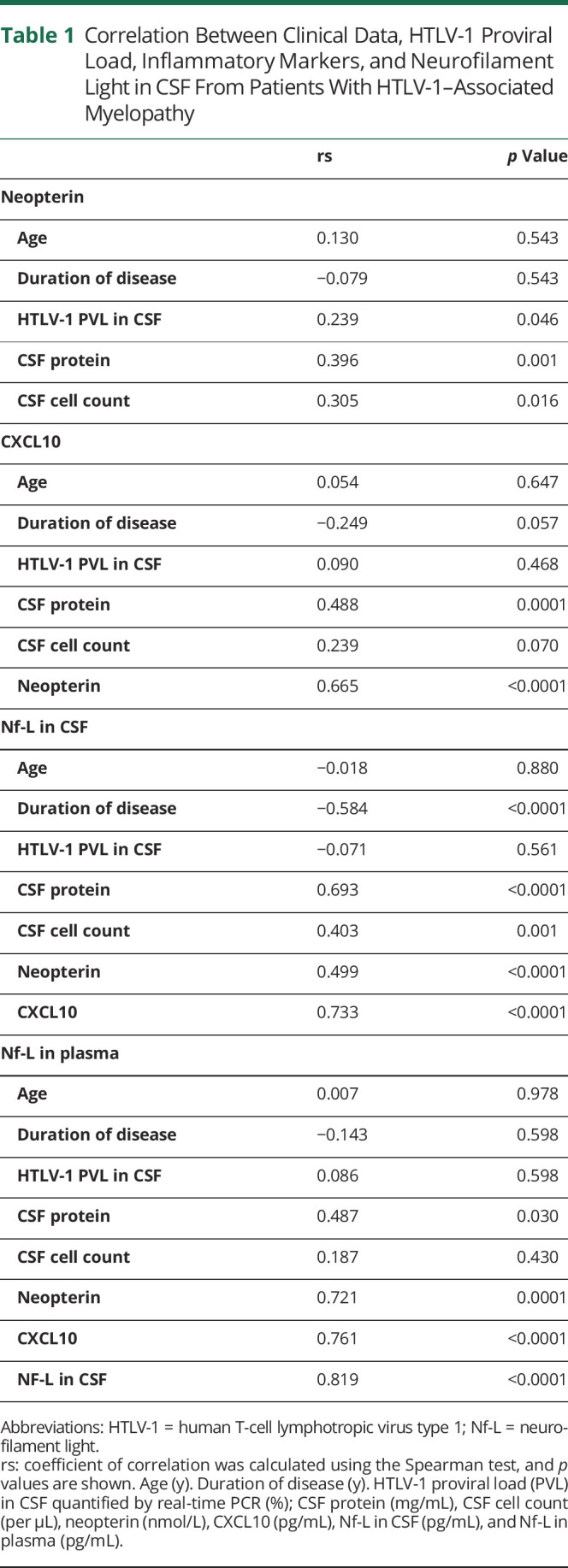
Correlation Between Clinical Data, HTLV-1 Proviral Load, Inflammatory Markers, and Neurofilament Light in CSF From Patients With HTLV-1–Associated Myelopathy

In patients with HAM, markers of inflammation (CXCL10 and neopterin) and neuronal damage (Nf-L) increased according to the degree of walking impairment being highest in wheelchair-dependent patients (Figure 1, D–F). There was no significant difference over time for all 3 CSF biomarkers (Figure 1, G–I). Of interest, high neopterin 77.03 nmol/L and CXCL10 6,532 pg/mL concentrations could be observed in a patient even 23 years after disease onset with 11 years of wheelchair dependency.

## Discussion

Neuronal damage in patients with HAM, evaluated by quantifying Nf-L in CSF and plasma, appears to be more intense early in the disease. This highlights the importance of early diagnosis and prompt intervention, which tends to have better results.^9^ However, persistent inflammation and neuronal damage was observed in most patients. With up to 11 years of follow-up between samples, this study shows that inflammatory markers remain elevated many years after disease onset. This evidence of ongoing CNS inflammation was also seen in wheelchair-dependent patients in whom clinical evidence of progression is difficult to detect. This suggests that even late in the disease course, some may still benefit from therapies that target inflammation, such as corticosteroids and steroid-sparing agents.^9^ Clinical studies would need long follow-up to prove benefit. Nf-L has the potential to evaluate continuing neurologic damage in patients with HAM and as a screening tool for therapeutic benefit. It can also be helpful as a tool for the stratification of patients into more homogenous groups for future clinical trials, as strongly recommended in the guideline for HAM treatment.^9^ Treatment for HAM is still limited, and clinical trials are urgently needed.

Recently, more sensitive techniques, such as SIMOA, have allowed Nf-L quantification in plasma samples, where the concentration is lower than CSF. Plasma sampling would make sequential sampling amenable both for patients with HAM and the follow-up of asymptomatic individuals. As Nf-L in plasma in this cohort of patients with HAM correlates not only with the levels of Nf-L in CSF but also with the CSF concentrations of inflammatory markers, it may be an alternative to monitor disease activity in patients with HTLV and to detect early, subclinical neuronal damage in those considered asymptomatic carriers of HTLV-1 infection. This would support therapeutic intervention even before clinical signs are evident, preventing or delaying HAM incidence and progression.

The present study has some limitations. Some patients were taking potentially disease-modifying therapy. In this center, the treatment is offered in a case-by-case manner, and those with more severe clinical presentation are those who are usually under treatment. No difference was observed between samples according to treatment status (data not shown). However, studies of the impact of treatment on Nf-L levels are important. Previous studies showed that inflammatory markers decrease with treatment and that this is associated with clinical outcome.^10^ Another limitation is the reference range for Nf-L. Although we used those proposed by the manufacturer, this is based on a limited number of samples and may be not truly representative.

In conclusion, neuroinflammation and neuronal damage, while being most intense early in disease, persists and is highest in those who have become wheelchair dependent. Nf-L has potential to be a useful biomarker in patients with HAM.

## Glossary

HAM: HTLV-1–associated myelopathy
HTLV-1: human T-cell lymphotropic virus type 1
Nf-L: neurofilament light
SIMOA: single molecule array
